# Comparative effectiveness of preoperative localization techniques for non-palpable breast lesions: multicentre real-world study

**DOI:** 10.1093/bjsopen/zraf153

**Published:** 2026-01-01

**Authors:** Fabio Corsi, Sara Albasini, Matilde Pelizzola, Carlo Morasso, Giulia Armatura, Alessandro Asaro, Corrado Chiappa, Virginia Coli, Francesca Combi, Angelica Della Valle, Raimondo Di Giacomo, Secondo Folli, Maria Luisa Gasparri, Massimo Maria Grassi, Stefano Mancini, Ilaria Maugeri, Marica Melina, Andrea Papadia, Lorenzo Rossi, Laura Roveda, Francesca Rovera, Silvia Segattini, Adele Sgarella, Claudio Siani, Norma Stefenelli, Francesco Valenti, Simone Zanotti

**Affiliations:** Department of Biomedical and Clinical Sciences, University of Milan, Milan, Italy; Breast Unit, Istituti Clinici Scientifici Maugeri IRCCS, Pavia, Italy; Breast Unit, Istituti Clinici Scientifici Maugeri IRCCS, Pavia, Italy; General Surgery Residency Program, University of Milan, Milan, Italy; Breast Unit, Istituti Clinici Scientifici Maugeri IRCCS, Pavia, Italy; Chirurgia Plastica e Senologica, Azienda Provinciale per i Servizi Sanitari di Trento, Trento, Italy; Breast Surgery Unit, Department of Surgery, ASST Fatebenefratelli—Sacco, Luigi Sacco University Hospital, Milano, Italy; SC Breast Unit, ASST-Settelaghi di Varese, Varese, Italy; UOSD Oncologia Chirurgica Ricostruttiva della Mammella, PO ‘F.Lotti’—USL Toscana Nordovest, Pontedera, Italy; Division of Breast Surgical Oncology, Department of Medical and Surgical, Maternal–Infantile and Adult Sciences, University Hospital of Modena, Modena, Italy; General Surgery 3- Breast Surgery, Department of Surgery, Fondazione IRCCS Policlinico San Matteo, Pavia, Italy; Divisione di Chirurgia Oncologica Senologica, Istituto Nazionale Tumori IRCCS Fondazione Pascale, Napoli, Italy; Breast Unit, Surgery, Fondazione IRCCS Istituto Nazionale dei Tumori, Milan, Italy; Department of Gynecology and Obstetrics, Ente Ospedaliero Cantonale (EOC), Ospedale Regionale di Lugano, Lugano, Switzerland; Faculty of Biomedicine, University of Italian Switzerland (USI), Lugano, Switzerland; Centro di Senologia della Svizzera Italiana (CSSI), Ospedale Italiano di Lugano, Lugano, Switzerland; Breast Unit, Humanitas Gavazzeni e Castelli, Bergamo, Italy; Breast Surgery Unit, Department of Surgery, ASST Fatebenefratelli—Sacco, Luigi Sacco University Hospital, Milano, Italy; Breast Unit, Surgery, Fondazione IRCCS Istituto Nazionale dei Tumori, Milan, Italy; Breast Unit, IRCCS Azienda Ospedaliero-Universitaria, Bologna, Italy; Department of Gynecology and Obstetrics, Ente Ospedaliero Cantonale (EOC), Ospedale Regionale di Lugano, Lugano, Switzerland; Faculty of Biomedicine, University of Italian Switzerland (USI), Lugano, Switzerland; Centro di Senologia della Svizzera Italiana (CSSI), Ospedale Italiano di Lugano, Lugano, Switzerland; Oncologia Medica, Istituto di Oncologia della Svizzera Italiana (IOSI), Bellinzona, Switzerland; UOSD Oncologia Chirurgica Ricostruttiva della Mammella, PO ‘F.Lotti’—USL Toscana Nordovest, Pontedera, Italy; SC Breast Unit, ASST-Settelaghi di Varese, Varese, Italy; Division of Breast Surgical Oncology, Department of Medical and Surgical, Maternal–Infantile and Adult Sciences, University Hospital of Modena, Modena, Italy; General Surgery 3- Breast Surgery, Department of Surgery, Fondazione IRCCS Policlinico San Matteo, Pavia, Italy; Divisione di Chirurgia Oncologica Senologica, Istituto Nazionale Tumori IRCCS Fondazione Pascale, Napoli, Italy; Chirurgia Plastica e Senologica, Azienda Provinciale per i Servizi Sanitari di Trento, Trento, Italy; Breast Unit, Humanitas Gavazzeni e Castelli, Bergamo, Italy; Breast Unit, IRCCS Azienda Ospedaliero-Universitaria, Bologna, Italy

**Keywords:** breast-conserving surgery, wire-guided localization, radio-guided occult lesion localization, magnetic seed localization, carbon localization, surgical margins

## Abstract

**Background:**

The increasing detection of non-palpable breast lesions has made accurate preoperative localization essential to optimize breast-conserving surgery. Although multiple localization methods exist, there is still a lack of robust, large-scale, multicentre evaluations comparing different techniques.

**Methods:**

The LOCALIZATION01 study compares real-world data from 13 breast units in Italy and Switzerland on the impact of localization techniques on breast-conserving surgery for non-palpable lesions between 2016 and 2024. Four localization techniques were compared: wire-guided (WGL), radio-guided (ROLL), magnetic seed (MSL), and carbon (CL). The main outcomes were margin status, calculated resection ratio, postoperative complications, and surgical time. Subgroup analyses were performed for body mass index, lesion morphology and histopathology.

**Results:**

In total, 3241 patients were enrolled (ROLL 985, MSL 592, WGL 1079, and CL 585). ROLL achieved the highest rate of negative surgical margins, significantly outperforming MSL, WGL, and CL (97.5% *versus* 94.7% *versus* 94.5% *versus* 90.6%, respectively; *P* < 0.05). CL was associated with the highest postoperative complications rate (16.7%) *versus* ROLL (4.1%), MSL (4.5%), and WGL (2.1%) (*P* < 0.0001). The surgical time for MSL was significantly shorter when compared with WGL (46 *versus* 70 minutes (min); *P* < 0.0001) and CL (55 min; *P* < 0.0001). WGL had the most favourable calculated resection ratio (2.4), followed by MSL (2.6), ROLL (2.7), and CL (3.0). Multivariable analysis identified CL as an independent predictor of positive margins (odds ratio 1.82; *P* = 0.004), whereas ROLL was protective (odds ratio 0.45; *P* = 0.009).

**Conclusion:**

ROLL and MSL outperformed WGL and CL across multiple endpoints. CL data revealed objective limitations that suggest caution in its use. A personalized approach considering lesion morphology, body mass index, and logistics is recommended.

## Teaser

- Comparison of four localization techniques for non-palpable breast lesions.
- ROLL showed the highest rate of negative surgical margins.
- Multicentre study evaluates localization techniques in breast-conserving surgery.

## Introduction

The advancement of imaging technologies and the widespread implementation of mammographic screening programmes have led to a significant increase in the detection of non-palpable breast lesions^1,2^. In this context, a critical determinant for the success of breast-conserving surgery (BCS) is the achievement of negative surgical margins^3,4^. Intraoperative difficulties in identifying the exact location and extent of non-palpable lesions may lead to either excessive resections or inadequate excisions requiring reintervention^5,6^ with poorer aesthetic outcomes, psychological distress, delays in adjuvant treatments, and increased healthcare costs^7^. For these reasons, different preoperative localization techniques have been developed to improve lesion targeting and optimize the balance between complete excision and tissue conservation^7–10^.

Wire-guided localization (WGL), introduced in the 1970s, remains the most used technique^11–14^. Although WGL is considered the standard of care due to its advantages—being relatively inexpensive, accurate, easy to insert, and supported by the high level of radiologist expertise—it also has several limitations, including same-day placement, coordination among clinical teams, potential wire displacement, patient discomfort, and aesthetic concerns^11,12,15^.

To overcome these challenges, various alternative localization methods have been introduced in surgical practice. Radio-guided occult lesion localization (ROLL) involves the injection of a radiotracer (technetium-99m) into the lesion, allowing intraoperative detection via a gamma probe^16,17^. Carbon localization (CL) uses the ultrasound-guided injection of a sterile activated carbon suspension that leaves a visible track lasting several days^18–20^. Magnetic seed localization (MSL) is one of the most recent methods, using paramagnetic markers (for example Magseed^®^ or Sirius Pintuition^®^), detectable during surgery using a magnetic probe^21–24^. Preliminary studies suggest lower re-excision rates with MSL compared with WGL, but further evidence is needed to assess its true impact on margin status^12,25–28^.

Other advanced methods, such as radioactive seed localization (RSL) and radar-based systems like SAVI SCOUT^®^, have shown promising results in the international literature. However, due to their limited use in Italy, they were not included in the present study^29,30^.

Whereas no localization method has demonstrated absolute superiority, wire-free techniques, particularly radio-guided and magnetic systems, are emerging as reliable alternatives^7,13,16,31^.

To date, most studies in the literature have been retrospective single-centre analyses, or direct comparisons with WGL. This had resulted in a notable lack of large-scale, multicentre studies evaluating multiple techniques.

To address this gap, a survey was conducted across different breast units in Italy and Switzerland to assess which localization techniques were routinely employed. Based on these data, a retrospective comparative analysis of the four most commonly used methods—ROLL, MSL, WGL, and CL—was performed focusing on their effectiveness in achieving negative margins, ability to calibrate surgical resection, expressed as the ratio between lesion size and excised tissue volume (calculated resection ratio, CRR), and impact on surgical time^13,30,32,33^.

## Methods

### Study type and design

In 2023, Istituti Clinici Scientifici Maugeri conducted a survey across Italian and Swiss centres to gain insight into the standard surgical practices for non-palpable breast lesions localization. Ten centres, out of fifteen tertiary centres invited, responded to the survey, covering the entire geography of Italy and including one centre in Switzerland. As shown in *Fig. S1*, data revealed that the localization techniques were distributed among ROLL, MSL, WGL, and CL (respectively, 31% *versus* 23% *versus* 23% *versus* 23%). No other methods were reported. These data reflect the findings of a broader survey conducted in 2023 by Senonetwork^34^, which highlighted the most commonly used localization techniques in Italian BU. Based on these results, a retrospective observational study (LOCALIZATION01) was designed considering these techniques and data were collected in accordance with a study protocol approved by the Ethical Committee (EC) of the coordinating institution (Institute’s approval: CE2776, ClinicalTrial.gov ID: NCT05942105) and by the EC of the participating centres. The study involved 12 Italian centres and one major breast unit in Switzerland. All patients underwent BCS, with or without associated axillary surgery, and with localization of non-palpable breast lesions using the techniques routinely employed at the centres. For each patient, clinical and medical history information were collected. To determine whether a surgical procedure achieved clear margins on the excised specimen, the widely accepted definitions of ‘no ink on tumour’ for invasive carcinoma and a 2 mm margin for *in situ* carcinoma^6^ were adopted. The CRR was used to assess the extent of excess tissue removal. It was defined as the ratio between the total resection volume—calculated as the ellipsoid volume based on the three resection dimensions—and the optimal resection volume—estimated as the spherical volume of the lesion with an additional 1 cm margin (*Fig. S2*)^33^. Patients with multifocal disease and those undergoing extensive oncoplastic breast-conserving procedures were also excluded from the study.

Among the main outcomes, postoperative complications were included. As shown in *Table S1*, these comprised hematoma, wound infection or dehiscence, and bleeding. These complications were postoperative and not directly related to the localization procedure itself. Nevertheless, the complication rates were analysed across localization methods to explore whether any indirect associations could be observed. In addition, postprocedural haematoma occurring after the localization procedure was evaluated separately as a postprocedural complication. Finally, reintervention rates due to margin involvement were analysed for each localization technique, as reintervention referred exclusively to re-excision procedures for positive margins.

### Patient population

Patients operated on between 2016 and 2024 were consecutively included in this study by each centre until the number of cases that the centre had agreed to contribute was reached. The inclusion criteria were female patients over 18 years old, with non-palpable breast lesion who underwent upfront BCS and whose lesions were diagnosed by core biopsy as B3/C3, B4/C4, or B5/C5. Patients who were a candidate for or who had undergone neoadjuvant chemotherapy, as well as those with multifocal disease or undergoing extensive oncoplastic BCS, were excluded from the study.

### Localization techniques

The WGL procedure involves the insertion of a wire into the breast under imaging guidance, either on the day of surgery or the day before, depending on the organizational protocols of each centre. The wire guides the surgeon to the target lesion^35–37^.

The MSL technique was developed to address WGL limitations^38–40^. Magnetic seeds (Magseed or Sirius Pintuition^®^)^41^, small biocompatible stainless steel implants, are placed under mammographic or ultrasound guidance to mark the lesion. A magnetic probe then detects the seed to guide surgical excision. The magnetic marker is placed several days or even weeks in advance (up to 30 days). The magnetic signal can generally be detected up to 3–4 cm from the skin surface.

ROLL involves the peritumoral injection of technetium-99m (Tc-99m)^42^ under ultrasound or mammographic guidance, either the day of surgery or the day before. During surgery, a gamma probe detects the Tc-99m signal, emitting both numeric and acoustic signals that assist the surgeon in precisely calibrating the extent of resection.

The CL method involves the intratumoral injection of activated carbon, which provides a visual guide for the surgeon to calibrate the surgical resection accurately^43^. It can be performed days before surgery, as the carbon suspension remains stable within the tissue and easily identifiable during surgery.

*Table S2* reports the analytical comparison between the previous localization methods. The techniques were performed in the same manner across all centres. Each centre contributed with techniques regarded as the standard at their institution, widely implemented and supported by their expertise; therefore, each centre contributed with one or two methods at most. *Table S3* provides comprehensive details for the participating centres, including hospital type, activity volumes, localization methods employed, and the number of patients contributing to the study.

### Statistical analysis

A missing data analysis was performed to assess data completeness, with missing values defined as blank cells in the study database. The proportion of blank cells across the entire analysis data set was calculated and verified to be low and acceptable. Missing values were not imputed; each patient contributed the information available for their case. Variables were presented as medians, interquartile ranges (i.q.r.) and ranges, or as absolute numbers and percentages. Categorical variables were compared using the χ² test or Fisher’s exact test, as appropriate, whereas continuous variables were assessed using a pairwise two-sided multiple comparison analysis (Dwass, Steel, Critchlow–Fligner Method). To assess the potential impact of each variable significantly associated with surgical margin involvement following breast surgery, a logistic regression model was employed. Additionally, to evaluate whether the hospitals’ practices influenced the rate of margin involvement, a hierarchical logistic regression model with a random effect for the centre was performed. This analysis was necessary because each centre used the localization method they performed best with, which is clinically appropriate. However, this evaluation was conducted to ensure that this did not introduce bias, and to confirm that the performance of the localization methods was genuinely assessed rather than differences between centres outcomes. Statistical significance was defined as *P* < 0.05 (two-tailed). Data analysis was performed using SAS/STAT^®^ software (v. 9.4, SAS Institute Inc., Cary, USA).

## Results

### Characteristics of non-palpable lesions cohort by localization technique

A total of 3241 patients with non-palpable breast lesions undergoing BCS were included in the study. Data completeness was high (94%). Demographic and clinical characteristics are summarized in *Table 1*, with corresponding *P*-values for comparison in *Table S4*. In this study, WGL was used for 1079 patients, whereas a total of 592 patients were localized using MSL with successful clip retrieval in all cases. Additionally, 985 patients underwent localization using the WGL technique and CL was performed for 585 patients.

**Table 1:** Baseline characteristics of the entire cohort

	ROLL *n* = 985	MSL *n* = 592	WGL *n* = 1079	CL *n* = 585
Age at diagnosis (years), median (i.q.r., range)	57 (68-49, 21–91)	60 (70-51, 20–88)	63 (71-54, 30–91)	61 (69-51, 35–82)
**Hormonal status**				
Fertile	227 (26.7%)	119 (23.3%)	177 (16.4%)	153 (26.2%)
Menopause	623 (73.3%)	392 (76.7%)	902 (83.6%)	431 (73.8%)
**BMI**				
< 30	789 (90.5%)	286 (84.4%)	927 (85.9%)	348 (73.9%)
≥ 30	83 (9.5%)	53 (15.6%)	152 (14.1%)	123 (26.1%)
**Lesion morphology**				
Nodule	459 (47.3%)	344 (62.4%)	716 (66.4%)	314 (54%)
Microcalcification	442 (45.6%)	154 (28%)	277 (25.6%)	205 (35.2%)
Distorsion	69 (7.1%)	53 (9.6%)	86 (8%)	63 (10.8%)
Lesion size on imaging (mm), median (i.q.r., range)	10 (14-6, 1–120)	9 (12-7, 1–80)	10 (15-8, 1–120)	10 (15-7, 2–80)
**Cyto/histological result (core biopsy)**				
B3/C3	273 (27.9%)	91 (15.5%)	24 (2.2%)	46 (7.9%)
B4/C4	13 (1.3%)	10 (1.7%)	16 (1.5%)	12 (2.1%)
B5a/B5b/C5	692 (70.8%)	485 (82.8%)	1039 (96.3%)	527 (90%)
**Previous breast lesion**				
No	692 (81.6%)	397 (79.6%)	980 (90.8%)	525 (89.9%)
Yes	156 (18.4%)	102 (20.4%)	99 (9.2%)	59 (10.1%)
**Postprocedural haematoma**				
No	827 (97.6%)	507 (97.1%)	1057 (98%)	536 (95.9%)
Yes	20 (2.4%)	15 (2.9%)	22 (2%)	23 (4.1%)
**Surgeon who performed resection**				
Attending surgeon	625 (83.9%)	388 (91.5%)	961 (91.3%)	552 (95%)
Resident	120 (16.1%)	36 (8.5%)	91 (8.7%)	29 (5%)
**Surgical radicalization**				
No	504 (51.2%)	170 (28.7%)	461 (42.7%)	68 (11.6%)
Yes	481 (48.8%)	422 (71.3%)	618 (57.3%)	517 (88.4%)
**Type of axillary surgery**				
None	461 (46.9%)	176 (29.8%)	196 (18.2%)	176 (30.1%)
SLNB only	473 (48.1%)	384 (65.1%)	816 (75.6%)	382 (65.3%)
ALND	50 (5.1%)	30 (5.1%)	67 (6.2%)	27 (4.6%)
**pT**				
No (benign lesion)	224 (23.7%)	72 (13.1%)	6 (0.5%)	26 (4.5%)
pT1	465 (49.2%)	373 (68.1%)	774 (72.1%)	356 (62.2%)
pT1mic	26 (2.8%)	8 (1.5%)	17 (1.6%)	11 (1.9%)
pT2	35 (3.7%)	6 (1.1%)	59 (5.5%)	20 (3.5%)
pT3	0 (0%)	0 (0%)	1 (0.1%)	0 (0%)
pT4	0 (0%)	0 (0%)	0 (0%)	1 (0.2%)
pTIS	145 (18.7%)	66 (14.1%)	217 (20.2%)	158 (27.6%)
Hospitalization (days), median (i.q.r., range)	1 (2-1, 0–7)	2 (3-1, 0–10)	2 (3-2, 0–9)	2 (2-2, 0–6)

Values are *n* (%) unless otherwise indicated. ROLL, radio-guided occult lesion localization; MSL, magnetic seed localization; WGL, wire-guided localization; CL, carbon localization; i.q.r., interquartile range; BMI, body mass index; SLNB, sentinel lymph-node biopsy; ALND, axillary lymph-nodes dissection, pT, pathologic tumour.

Patients treated with WGL were the oldest (median age 63 (i.q.r. 71-54, range 30–91)), significantly older than with ROLL (57 (68-49, 21–91); *P* < 0.0001), MSL (60 (70-51, 20–88); *P* = 0.04), and CL (61 (69-51, 35–82); *P* = 0.01). The majority of patients were postmenopausal, particularly in the WGL group (83.6%), significantly higher than in the ROLL (73.3%; *P* < 0.0001), CL (73.8%; *P* < 0.0001) and MSL (76.7%; *P* = 0.001) groups.

There were more obese patients (body mass index (BMI) ≥ 30) in the CL group (26.1%) than in the ROLL (9.5%; *P* < 0.0001), MSL (15.6%; *P* = 0.0004), and WGL (14.1%; *P* < 0.0001) groups.

Regarding lesion morphology, nodules were more frequently localized with WGL (66.4%) compared with ROLL (47.3%; *P* < 0.0001) and CL (54%; *P* < 0.0001). Microcalcifications were more often found with ROLL (45.6%) compared with MSL (28%; *P* < 0.0001), WGL (25.6%, *P* < 0.0001), and CL (35.2%; *P* < 0.0001). CL was associated with the highest proportion of distortions (10.8%), higher than ROLL (7.1%, *P* < 0.0001), MSL (9.6%; *P* = 0.01), and WGL (8%; *P* < 0.0001).

Median lesion size was similar between ROLL and MSL (10 mm (i.q.r. 14-6, range 1–120) *versus* 9 mm (12-7, 1–80); *P* = 0.17) and between WGL and CL (both 10 mm (i.q.r. 15-8 and 15-7, respectively); *P* = 1.00), although the size distribution differed significantly between the two pairs (*P* < 0.05), these differences did not change the pathologic tumour (pT) size category according to the tumour, node, metastasis (TNM) staging system, as all median lesion sizes for all localization techniques fell within the pT1 category.

The proportion of histologically malignant lesions (B5a/B5b/C5) was higher in WGL (96.3%) and CL (90%) than in MSL (82.8%) and ROLL (70.8%); *P* < 0.05. Additional analyses are reported below to evaluate whether these differences influenced margin status across the various localization techniques.

### Outcomes under investigation

**Fig. 1:**
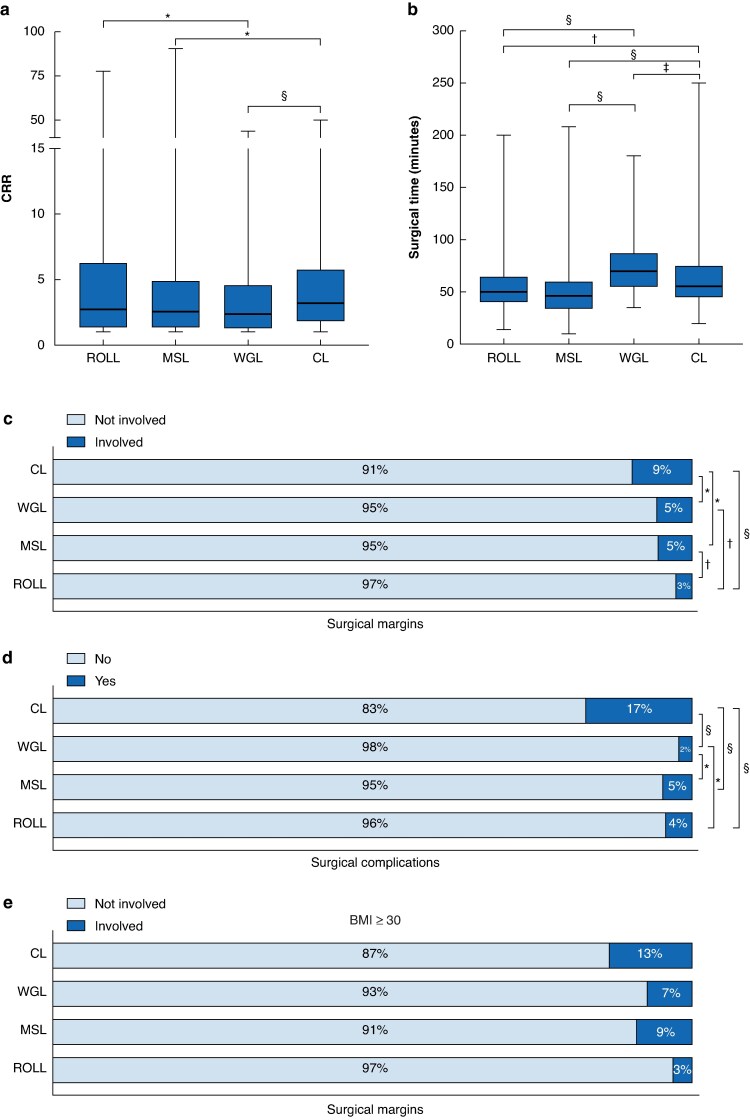
Comparison of localization techniques across surgical outcomes **a** Clinical resection ratio (CRR) (*P < 0.02, §*P* < 0.0001); **b** operative time for simple breast-conserving surgery (†*P* = 0.002, ‡*P* = 0.0004, §*P* < 0.0001); **c** surgical margin involvement (*P < 0.04, †*P* < 0.0008, §*P* < 0.0001); **d** postoperative complication rates (*P < 0.008, §*P* < 0.0001); and **e** margin involvement in patients with BMI ≥ 30 (*P* = 12). ROLL, radio-guided occult lesion localization; MSL, magnetic seed localization; WGL, wire-guided localization; CL, carbon localization.

According to *Table 2*, *Table S5*, and *Fig. 1*, ROLL achieved the highest rate of negative margin (97.5%), which was significantly higher than MSL (94.7%; *P* = 0.001), WGL (94.5%; *P* = 0.0008), and CL (90.6%; *P* < 0.0001). MSL and WGL were equivalent (*P* = 0.85), whereas CL was significantly worse than all other methods. These findings were also confirmed in the lesions classified as malignant at core biopsy (*Table S6*).

**Table 2.** Outcomes under investigation

	ROLL	MSL	WGL	CL
**All patients**				
Surgical margins				
Not involved	956 (97.5%)	558 (94.7%)	1018 (94.5%)	528 (90.6%)
Involved	25 (2.6%)	31 (5.3%)	59 (5.5%)	55 (9.4%)
CRR, median (i.q.r., range)	2.7 (6.2-1.3, 1–77.7)	2.6 (4.9-1.4, 1–90.7)	2.4 (4.6-1.3, 1–43.7)	3 (5.3-1.7, 1–50)
Surgical complications				
No	907 (95.9%)	557 (95.5%)	1054 (97.9%)	485 (83.3%)
Yes	39 (4.1%)	26 (4.5%)	23 (2.1%)	97 (16.7%)
**Surgery time (min), median (i.q.r., range)**				
BCS only	50 (65-40, 14–200)	46 (60-34, 10–208)	70 (87-55, 35–180)	55 (75-450, 20–250)
**BMI ≥ 30**				
Surgical margins not involved	64 (97%)	42 (91.3%)	133 (93%)	100 (87%)
Surgical margins involved	2 (3%)	4 (8.7%)	10 (7%)	15 (13%)

Values are *n* (%) unless otherwise indicated. ROLL, radio-guided occult lesion localization; MSL, magnetic seed localization; WGL, wire-guided localization; CL, carbon localization; CRR, calculated resection ratio; i.q.r., interquartile range; min, minutes; BCS, breast-conserving surgery; BMI, body mass index.

WGL was associated with a lower and more favourable CRR (median 2.4 (i.q.r. 4.6-1.3, range 1–43.7)) than ROLL (2.7 (6.2-1.3, 1–77.7); *P* = 0.01). The CRR in MSL was lower than in CL (2.6 (4.9-1.4, 1–90.7) *versus* 3 (5.3-1.7, 1–50), respectively; *P* = 0.02), but not significantly different from that in ROLL or WGL. CL showed the highest CRR (3 (5.3-1.7, 1–50)), with significant differences compared with both MSL (*P* = 0.02) and WGL (*P* < 0.0001).

The postoperative complication rate was higher in CL (16.7%) than in ROLL (4.1%), MSL (4.5%), and WGL (2.1%); *P* < 0.0001. WGL had the lowest complication rate, which was statistically significant. Regarding postprocedural haematoma, CL was associated with the highest rate (4.1%), significantly higher than ROLL (2.4%; *P* = 0.06) and WGL (2.0%; *P* = 0.01), but not significantly different from MSL (2.9%; *P* = 0.27).

Analysing the surgical time, MSL was the fastest technique (min) for BCS only (median 46 (i.q.r. 60-34, range 10–208)), followed by WGL (70 (87-55, 35–180); *P* < 0.0001) and CL (55 (75-45, 20–250); *P* < 0.0001).

Stratifying by BMI, no association was found in patients with BMI ≥ 30 (*P* = 0.12). However, there is a trend indicating that ROLL provides better margin clearance (3%) than WGL (7%) and MSL (8.7%). All three techniques performed better than CL (13%).

Some other outcomes are reported in *Tables S7* and *S8*: CL showed the highest (10.9%) reintervention rate for involved margins, significantly higher than ROLL (3.7%; *P* < 0.0001), MSL (4.3%; *P* < 0.0001), and WGL (7%; *P* = 0.006). For the patient group that had undergone BCS with sentinel lymph node biopsy (SLNB), MSL remained the fastest (min) technique (median 57 (i.q.r. 70-45, range 18–180)), followed by ROLL (60 (72-50, 25–189); *P* = 0.01), CL (75 (95-55, 19–180); *P* < 0.0001), and WGL (95 (115-77, 40–225); *P* < 0.0001). In BCS with axillary dissection, MSL showed the lowest duration (87 (114-60, 30–180)), and was significantly shorter than WGL (143 (180-100, 95–218); *P* = 0.01).

### Correlation between surgical margin involvement, localization technique, and different features

After excluding uncertain lesions (B3/B4) and focusing on malignant cases (B5a/B5b/C5) where the margins status is critical, it was observed that margin involvement significantly differed according to lesion morphology and localization technique (*Table S9*). In nodular lesions, ROLL was associated with the lowest rate of margin positivity (1%), compared with MSL (4%), WGL (4.3%), and CL (8.5%) (*P* < 0.0001). ROLL also showed the lowest microcalcifications rate (5.8%), compared with MSL (9.5%), WGL (8.5%), and CL (14.5%) (*P* = 0.03). In contrast, for distortions, no significant association was found between technique and margin involvement (*P* = 0.89).

### Calculated resection ratio analysis

An in-depth analysis of CRR stratified by histology is presented in *Table S10*. In invasive carcinomas, CL had the highest CRR (median 3 (i.q.r. 5.2-1.9, range 1–40.3)), significantly higher than MSL (2.4 (4.3-1.4, 1–60.8); *P* = 0.002), WGL (2.5 (4.7-1.5, 1–43.7); *P* < 0.0001), and ROLL (2.7 (5.6-1.5, 1–60.3); *P* = 0.02). MSL and WGL were equivalent (*P* = 1.00). In *in situ* carcinomas, WGL showed the most favourable median value (1.5 (3.6-1, 1–17.1)), significantly better than ROLL (2.9 (8.3-1.1, 1–72.3); *P* = 0.009) and CL (2.5 (5.2-1.2, 1–36.5); *P* = 0.009). MSL and WGL did not differ significantly (*P* = 0.19). Among uncertain lesions, the trend favoured CL, though not significantly.

Stratifying by BMI, in patients with BMI < 30, MSL had the lowest CRR (median 1.8 (i.q.r. 3.2-1.1, range 1–14.8)), significantly better than ROLL (2.7 (6.5-1.4, 1–77.7); *P* < 0.0001) and CL (2.7 (4.8-1.5, 1–40.3); *P* < 0.0001), but not WGL (2.1 (4-1.2, 1–43.7); *P* = 0.08). In BMI ≥ 30 patients, WGL had the highest CRR (4.3 (7.5-2.5, 1–30)), but differences were not statistically significant.

For nodular lesions, CL had the worst CRR (median 2.9 (i.q.r. 5-1.7, range 1–36.5)), significantly higher than ROLL (2.2 (3.9-1.2, 1–77.8)), MSL (2 (3.6-1.3, 1–29.6)), and WGL (2.4 (4.3-1.4, 1–21.5)); *P* < 0.05. In microcalcifications, WGL showed the best performance (2.5 (5.8-1, 1–31.6)), with higher CRRs in ROLL (4 (9.2-1.8, 1–72.3)), MSL (4.3 (7.8-2.2, 1–90.7)), and CL (3.3 (5.9-2.5, 1–49.9)); *P* < 0.05). Differences among the latter three were not significant. In distortions, no overall difference emerged except for WGL *versus* CL (1.8 (3.6-1.2, 1–43.7) *versus* 3.1 (5.2-2.1, 1–22), respectively; *P* = 0.02).

### Multivariable analysis

The multivariable logistic regression model, shown in *Table 3*, identified CL as an independent predictor of margin positivity (odds ratio (OR) 1.82, 95% confidence interval (c.i.) 1.22 to 2.73; *P* = 0.004) and ROLL had a protective effect (OR 0.45, 0.25 to 0.82; *P* = 0.009) compared with WGL. MSL did not significantly differ from WGL (*P* = 0.90). Microcalcifications (OR 1.99, 1.14 to 3.45; *P* = 0.01) and distortions (OR 1.93, 1.29 to 2.90; *P* = 0.001) were associated with increased risk of incomplete excision compared with nodules.

**Table 3:** Multivariable analysis

	Computed the probability of surgical margins involvement		
	OR	95% c.i.	P*
**Localization technique**			
CL	1.82	1.22, 2.73	0.004
MSL	1.04	0.59, 1.83	0.90
ROLL	0.45	0.25, 0.82	0.009
WGL	Ref.		
**Surgeon who performed resection**			
Attending surgeon	1.66	0.76, 3.63	0.21
Resident	Ref.		
**Lesion morphology**			
Microcalcification	1.99	1.14, 3.45	0.01
Distorsion	1.93	1.29, 2.9	0.001
Nodule	Ref.		
Lesion size on imaging (mm)	1.01	1, 1.03	0.05
CRR	0.99	0.95, 1.02	0.47

OR, odds ratio; c.i., confidence interval; Ref., reference cathegory; CL, carbon localization; MSL, magnetic seed localization; ROLL, radio-guided occult lesion localization; WGL, wire-guided localization; CRR, calculated resection ratio. *Wald Chi-Square.

A hierarchical model with random effect for centre showed no significant centre effect (*P* ≥ 0.05); no *P*-value was computed for two small centres (< 41 cases) (*Table S11*).

Results were confirmed even after excluding uncertain lesions (B3/B4), as shown in *Table S12*.

## Discussion

Although WGL is the most widely used method, it has several drawbacks, including same-day placement, challenging team coordination, wire displacement, patient discomfort, and cosmetic concerns^8,9^. In this context, new methods have been developed, and others are currently being proposed and implemented, aiming to improve resection performance^9^.

To date, the literature primarily reports comparative data of individual techniques *versus* WGL, mostly from retrospective, single-centre, and relatively small case series. Here, the four most widely used localization techniques were compared in a multicentre setting.

Regarding the overall margin infiltration rate across all lesion types, data showed that ROLL performs significantly better than WGL, confirming the data present in the literature^35,44,45^, and MSL, whereas CL shows significantly inferior performance.

Margin infiltration rates by lesion morphology revealed that distortions are the most challenging, with similar performance across techniques. For microcalcifications and nodules, ROLL significantly outperformed WGL and MSL (which are equivalent) and was markedly superior to CL. This result is strengthened by the fact that the significant difference emerged in the context of B5a/B5b/C5 lesions only, with uncertain lesions excluded. These results are consistent with those of the COBALT study^46^, which highlighted the difficulty in achieving complete resections in the presence of distortions.

In terms of CRR, ROLL, WGL, and MSL overall showed similar performance, all outperforming CL. When focusing on invasive lesions, the results confirmed the general comparison: all techniques are equivalent except for CL, which performs worse. For *in situ* lesions, WGL appeared to be the best method, comparable to MSL, and superior to ROLL and CL.

The analysis of CRR by lesion morphology revealed a substantial equivalence among techniques for distortions (which remain the most complex lesions in terms of both localization and radicality of resection), confirming the results seen on the margin status. For microcalcifications and nodules, all techniques perform similarly except for CL, which was significantly inferior.

An interesting aspect of these data concerns performance in relation to patient BMI. It is well known that obesity correlates with increased breast adipose tissue, complicating the detection of non-palpable lesions^47,48^. Data showed that CRR is comparable across techniques in patients with a BMI ≥ 30. However, there was a trend suggesting that ROLL yields superior margin clearance compared with WGL and MSL, which are similar, and all three outperform CL.

These findings were confirmed by multivariable analysis, accounting for surgeon experience (attending and resident), lesion size and morphology, and excluding benign lesions and B3/B4 lesions.

Another objective of the study was to assess how different localization methods impact surgical time. In BCS without axillary intervention, MSL and ROLL showed equivalent times, both significantly shorter than CL and WGL. Including SLNB, the trend was confirmed. These results corroborate the literature^49^ that reported reduced operative times for MSL compared with WGL.

Postoperative complication rates were similar across all techniques except for CL, where they were significantly higher. Previous studies^9,50^ have also raised concerns about an higher risk of complications associated with CL. Hospital stay durations were not considered due to organizational differences among the participating centres, which strongly influence this variable.

Overall, data do not demonstrate a clear superiority of one technique over others, although ROLL and MSL appeared to perform generally better in terms of margin infiltration rates, CRR, and operative times. CL data revealed objective limitations that should suggest caution in its use. Nevertheless, this technique is used in several centres across Italy, where it represents a well-established alternative to wire-guided or radio-guided localization. In other European countries, its use appears to be more variable. The technique offers logistical simplicity and low cost, providing a reliable visual guide for lesion excision. It is evident that the choice of localization technique should consider costs, team familiarity, availability, and accessibility of related services, such as nuclear medicine for ROLL. Furthermore, having multiple localization options available could allow their optimal use in the most suitable clinical contexts, positively impacting patient outcomes.

Patient-reported outcomes on quality of life and data on surgeon satisfaction^34^ would represent a valuable addition to this work and could serve as the basis for future development of this study^51^. However, they were not included here as such data are not routinely or uniformly collected across the clinical centres involved. Finally, prospective studies, which are expected soon, will further support surgeons in making informed choices.

## Supplementary Material

zraf153_Supplementary_Data

## Data Availability

The data set generated and analysed during the present study is available from the corresponding author upon reasonable request.
