# Supplementary material for: Comparative effectiveness of preoperative localization techniques for non-palpable breast lesions: multicentre real-world study
Source: BJS Open. 2026 Jan 1;10(1):zraf153. doi: 10.1093/bjsopen/zraf153 (PMC12781200; doi:10.1093/bjsopen/zraf153)
Supplement: zraf153_Supplementary_Data [file zraf153_supplementary_data.docx]

**Comparative effectiveness of preoperative localization techniques for non-palpable breast lesions: multicenter real-world study**

Fabio Corsi^1,2^*, MD, Sara Albasini^2^, MsC, Matilde Pelizzola^3^, MsC, Carlo Morasso^2^, PhD, Giulia Armatura^4^, MD, PhD, Alessandro Asaro^5^, MD, Corrado Chiappa^6^, MD, Virginia Coli^7^, MD, Francesca Combi^8^, MD, Angelica Della Valle^9^, MD, Raimondo Di Giacomo^10^, MD, Secondo Folli^11^, MD, Maria Luisa Gasparri^12,13,14^, MD, PhD, Massimo Maria Grassi^15^, MD, Stefano Mancini^5^, MD, Marica Melina^16^, MD, Ilaria Maugeri^11^, MD, Andrea Papadia^12,13^, MD, PhD, Lorenzo Rossi^14,17^, MD, Laura Roveda^7^, MD, Francesca Rovera^6^, MD, Silvia Segattini^8^, MD, Adele Sgarella^9^, MD, Claudio Siani^10^, MD, Norma Stefenelli^4^, MD, Francesco Valenti^15^, MD, Simone Zanotti^16^ MD

1. Department of Biomedical and Clinical Sciences, University of Milan, Milan, Italy
2. Istituti Clinici Scientifici Maugeri IRCCS, Pavia, Italy
3. General Surgery Residency Program, University of Milan, Milan, Italy
4. Chirurgia Plastica e Senologica, Azienda Provinciale per i Servizi Sanitari di Trento, Italy
5. Breast Surgery Unit, Department of Surgery, ASST Fatebenefratelli - Sacco, Luigi Sacco University Hospital, Milano, Italy
6. SC Breast Unit, ASST-Settelaghi di Varese, Varese, Italy
7. UOSD Oncologia Chirurgica Ricostruttiva della Mammella, PO "F.Lotti" - USL Toscana Nordovest – Pontedera, Italy
8. Division of Breast Surgical Oncology, Department of Medical and Surgical, Maternal-Infantile and Adult Sciences, University Hospital of Modena, Modena, Italy
9. General Surgery 3‐ Breast Surgery, Department of Surgery, Fondazione IRCCS Policlinico San Matteo, Pavia, Italy
10. I.R.C.C.S. “G. Pascale”, Napoli, Italy
11. Breast Unit, Surgery, Fondazione IRCCS Istituto Nazionale dei Tumori, Milan, Italy
12. Dep. of Gynecology and Obstetrics, Ente Ospedaliero Cantonale (EOC), Ospedale Regionale di Lugano, Lugano, Switzerland
13. Faculty of Biomedicine, University of Italian Switzerland (USI), Lugano, Switzerland
14. Centro di Senologia della Svizzera Italiana (CSSI), Ospedale Italiano di Lugano, Lugano, Switzerland
15. Breast Unit, Humanitas Gavazzeni e Castelli, Bergamo, Italy
16. Breast Unit, IRCCS Azienda Ospedaliero-Universitaria, Bologna, Italy
17. Istituto di Oncologia della Svizzera Italiana (IOSI), Bellinzona, Switzerland

*Corresponding Author:

Fabio Corsi, MD

Istituti Clinici Scientifici Maugeri IRCCS, Pavia, Italy

Department of Biomedical and Clinical Sciences, University of Milan, Milan, Italy

E-mail: fabio.corsi@icsmaugeri.it - Phone: +39 0382592272

**Supplementary Materials - Index**

| **Supplementary Figures and Tables** |  |
| --- | --- |
| Supplementary Figure S1 | *page 3* |
| Supplementary Figure S2 | *page 3* |
| Supplementary Table S1 | *page 4* |
| Supplementary Table S2 | *page 4* |
| Supplementary Table S3 | *page 4* |
| Supplementary Table S4 | *page 5* |
| Supplementary Table S5 | *page 6* |
| Supplementary Table S6 | *page 6* |
| Supplementary Table S7 | *page 6* |
| Supplementary Table S8 | *page 7* |
| Supplementary Table S9 | *page 7* |
| Supplementary Table S10 | *page 8* |
| Supplementary Table S11 | *page 9* |
| Supplementary Table S12 | *page 9* |
| **References** | *page 10* |
|  |  |

**Supplementary Figures and Tables**

**Supplementary Figure S1** Results from survey on localization techniques for non-palpable breast lesions

**Supplementary Figure S2** Mathematical calculation of Calculated Resection Ratio (CRR) (1)

**
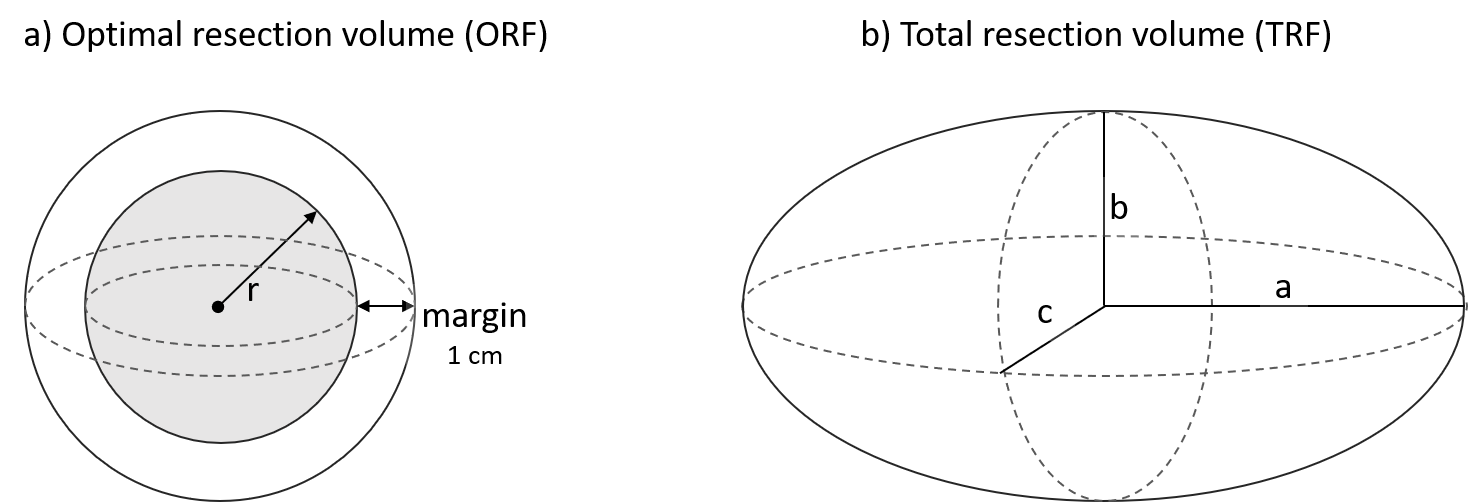
**

The tumour was defined as a sphere, and the tumour volume was calculated by the formula 4/3πr³. *r* represents the radius, which is equal to one half of the diameter measured by the pathologist.

(a) Optimal resection volume (ORV) was calculated for each tumour. ORV was defined as the spherical tumour volume with an added 1.0-cm margin of healthy breast tissue, calculated by the formula 4/3π(*r* + 1.0)³.

(b) Total resection volume (TRV) represents the volume of the surgical specimen. TRV was assumed to be an ellipsoid in shape and was calculated using the three dimensions of the surgical specimen (in cm) measured by the pathologist. The formula applied to calculate TRV was 4/3π(*a·b·c*), with *a*, *b*, and *c* representing one half of each of the three dimensions of the surgical specimen.

CRR was calculated as ratio TRV/ORV.

**Supplementary Table S1** Type of post-operative complications

| **Complication** | **ROLL** | **MSL** | **WGL** | **CL*** | **p** |
| --- | --- | --- | --- | --- | --- |
| **Bleeding** | 5 (12.8) | 2 (7.7) | 4 (17.4) | 10 (9.9) | 0.86 |
| **Hematoma** | 24 (61.5) | 18 (69.3) | 15 (65.2) | 60 (59.4) |  |
| **Wound infection** | 6 (15.4) | 3 (11.5) | 2 (8.7) | 12 (11.9) |  |
| **Dehiscence** | 4 (10.3) | 3 (11.5) | 2 (8.7) | 19 (18.8) |  |

Values are n (%). ROLL= Radioguided Occult Lesion Localization; MSL= Magnetic Seed; WGL= Wire Gide Localization; CL= Carbon Localization

*5 patients experienced 2 different complications

**Supplementary Table S2** Analytical comparison between the localization methods

| **Localization method** | **Device** | **Timing of placement** | **Intraoperative Detection** | **Maximum detectable depth** |
| --- | --- | --- | --- | --- |
| **WGL** | Metallic wire inserted into the lesion | Same day or day before surgery, depending on center logistics | Direct palpation and wire guidance | Not limited (wire visible intraoperatively) |
| **MSL** | Magnetic seed (e.g., Magseed®, Sirius Pintuition®) | Several days or weeks before surgery (up to 30 days) | Magnetic probe detecting the seed signal | ~3–4 cm from skin surface |
| **ROLL** | Peritumoral injection of Technetium-99m (Tc-99m) | Day of or day before surgery (depending on radioprotection protocols) | Gamma probe emitting numeric and acoustic feedback | Not limited (radiotracer-based detection) |
| **CL** | Intratumoral injection of carbon suspension | Several days before surgery | Direct visual identification of black-stained tissue | Not limited (visual detection) |

ROLL= Radioguided Occult Lesion Localization; MSL= Magnetic Seed; WGL= Wire Gide Localization; CL= Carbon Localization

**Supplementary Table S3** Main information on participating centers

| **Center** | **Type** | **N° primary breast cancers treated per annum** | **Localization method** | **ROLL** | **MSL** | **WGL** | **CL** | **Total** |
| --- | --- | --- | --- | --- | --- | --- | --- | --- |
| **Istituti Clinici Scientifici IRCCS - Pavia** | RH | 450 | ROLL, MSL | 298 | 128 | - | - | 426 |
| **IRCCS Azienda Ospedaliero-Universitaria - Bologna** | UH | 515 | ROLL, MSL | 370 | 126 | - | - | 496 |
| **ASST Sette Laghi - Varese** | UH | 545 | ROLL | 150 | - | - | - | 150 |
| **Ente Ospedaliero Cantonale - Svizzera** | UH | 250 | WGL | - | - | 654 | - | 654 |
| **Humanitas Gavazzeni - Bergamo** | APH | 330 | ROLL | 167 | - | - | - | 167 |
| **Istituto Nazionale dei Tumori - Milano** | RH | 800 | MSL | - | 200 | - | - | 200 |
| **I.R.C.C.S. Fondazione G. Pascale - Napoli** | RH | 460 | MSL | - | 97 | - | - | 97 |
| **ASST Fatebenefratelli Sacco - Milano** | UH | 250 | WGL | - | - | 26 | - | 26 |
| **Azienda Ospedaliero Universitaria - Modena** | UH | 780 | CL | - | - | - | 363 | 363 |
| **Fondazione IRCCS Policlinico San Matteo - Pavia** | RH | 340 | WGL | - | - | 399 | - | 399 |
| **PO "F.Lotti" - USL Toscana Nordovest - Pontedera** | LHU | 220 | CL | - | - | - | 122 | 122 |
| **Azienda Provinciale per i Servizi Sanitari - Trento** | LHU | 550 | CL | - | - | - | 100 | 100 |
| **Azienda Sanitaria n.1 Regione Umbria - Città di Castello** | LHU | 330 | MSL | - | 41 | - | - | 41 |
| **Total** |  |  |  | 985 | 592 | 1079 | 585 | **3241** |

UH= University hospital; APH= Accredited private hospital; RH= Research hospital of national interest; LHU= Local health unit; ROLL= Radioguided Occult Lesion Localization; MSL= Magnetic Seed; WGL= Wire Gide Localization; CL= Carbon Localization

**Supplementary Table S4** Comparisons (p-values) for each method and variable related to Table 1

|  | **Age** | | | **Hormonal status** | | | **BMI** | | | **Lesion size on imaging** | | |
| --- | --- | --- | --- | --- | --- | --- | --- | --- | --- | --- | --- | --- |
|  | **MSL** | **WGL** | **CL** | **MSL** | **WGL** | **CL** | **MSL** | **WGL** | **CL** | **MSL** | **WGL** | **CL** |
| **ROLL** | 0.01 | <0.0001 | 0.003 | 0.16 | <0.0001 | 0.83 | 0.002 | 0.002 | <0.0001 | 0.17 | <0.0001 | 0.002 |
| **MSL** |  | 0.04 | 0.98 |  | 0.001 | 0.27 |  | 0.48 | 0.0004 |  | <0.0001 | <0.0001 |
| **WGL** |  |  | 0.01 |  |  | <0.0001 |  |  | <0.0001 |  |  | 1.00 |
|  | **Previous breast lesion** | | | **Lesion morphology** | | | **Cyto/histological result (CB)** | | | **Post-procedure hematoma** | | |
|  | **MSL** | **WGL** | **CL** | **MSL** | **WGL** | **CL** | **MSL** | **WGL** | **CL** | **MSL** | **WGL** | **CL** |
| **ROLL** | 0.36 | <0.0001 | <0.0001 | <0.0001 | <0.0001 | <0.0001 | <0.0001 | <0.0001 | <0.0001 | 0.56 | 0.63 | 0.06 |
| **MSL** |  | <0.0001 | <0.0001 |  | 0.25 | 0.01 |  | <0.0001 | 0.002 |  | 0.30 | 0.27 |
| **WGL** |  |  | 0.54 |  |  | <0.0001 |  |  | <0.0001 |  |  | 0.01 |
|  | **Surgeon who performed resection** | | | **Surgical radicalization** | | | **Type of axillary surgery** | | | **pT** | | |
|  | **MSL** | **WGL** | **CL** | **MSL** | **MSL** | **MSL** | **MSL** | **WGL** | **CL** | **MSL** | **WGL** | **CL** |
| **ROLL** | 0.0002 | <0.0001 | <0.0001 | <0.0001 | 0.0001 | <0.0001 | <0.0001 | <0.0001 | <0.0001 | <0.0001 | <0.0001 | <0.0001 |
| **MSL** |  | 0.92 | 0.03 |  | <0.0001 | <0.0001 |  | <0.0001 | 0.93 |  | <0.0001 | <0.0001 |
| **WGL** |  |  | 0.007 |  |  | <0.0001 |  |  | <0.0001 |  |  | <0.0001 |
|  | **Hospitalization** | | |  | | |  | | |  | | |
|  | **MSL** | **WGL** | **CL** |  |  |  |  |  |  |  |  |  |
| **ROLL** | <0.0001 | <0.0001 | <0.0001 |  |  |  |  |  |  |  |  |  |
| **MSL** |  | 0.03 | 0.33 |  |  |  |  |  |  |  |  |  |
| **WGL** |  |  | <0.0001 |  |  |  |  |  |  |  |  |  |

ROLL= Radioguided Occult Lesion Localization; MSL= Magnetic Seed; CB= core biopsy; WGL= Wire Gide Localization; CL= Carbon Localization; CB= core biopsy; BMI= Body mass index.

**Supplementary Table S5** Comparisons (p-values) for each method and variable related to Table 2 and Figure 1

|  | **Surgical margins** | | | **CRR** | | |
| --- | --- | --- | --- | --- | --- | --- |
|  | **MSL** | **WGL** | **CL** | **MSL** | **WGL** | **CL** |
| **ROLL** | 0.001 | 0.0008 | <0.0001 | 0.59 | 0.01 | 0.36 |
| **MSL** |  | 0.85 | 0.04 |  | 0.56 | 0.02 |
| **WGL** |  |  | 0.02 |  |  | <0.0001 |
|  | **Surgical complications** | | | **Surgery time (BCS only)** | | |
|  | **MSL** | **WGL** | **CL** | **MSL** | **WGL** | **CL** |
| **ROLL** | 0.75 | 0.01 | <0.0001 | 0.09 | <0.0001 | 0.002 |
| **MSL** |  | 0.008 | <0.0001 |  | <0.0001 | <0.0001 |
| **WGL** |  |  | <0.0001 |  |  | 0.0004 |

ROLL= Radioguided Occult Lesion Localization; MSL= Magnetic Seed; CB= core biopsy; WGL= Wire Gide Localization; CL= Carbon Localization; CRR= calculated resection ratio; BCS= Breast-conserving surgery.

**Supplementary Table S6** Surgical margins for B5a/B5b/C5 lesions only and relative p-values

| **Characteristics** | | **ROLL**  **n=691** | **MSL**  **n=483** | **WGL**  **n=1037** | | **CL**  **n=526** |
| --- | --- | --- | --- | --- | --- | --- |
| **Surgical margins** | |  |  |  | |  |
| Not involved | | 669 (96.8) | 456 (94.4) | 978 (94.3) | | 472 (89.7) |
| Involved | | 22 (3.2) | 27 (5.6) | 59 (5.7) | | 54 (10.3) |
|  | **p-values** | | | |  |  |
|  | **MSL** | **WGL** | **CL** | |  |  |
| **ROLL** | 0.04 | 0.02 | <0.0001 | |  |  |
| **MSL** |  | 0.94 | 0.007 | |  |  |
| **WGL** |  |  | 0.001 | |  |  |

Values are n (%). ROLL= Radioguided Occult Lesion Localization; MSL= Magnetic Seed; CB= core biopsy; WGL= Wire Gide Localization; CL= Carbon Localization.

**Supplementary Table S7** Reinterventions for involved margins and relative p-values

| **Characteristics** | | | **ROLL** | **MSL** | **WGL** | **CL** |
| --- | --- | --- | --- | --- | --- | --- |
| **Reinterventions** | | |  |  |  |  |
| No | | | 942 (96.3) | 560 (95.7) | 1003 (93) | 516 (89.1) |
| Yes | | | 36 (3.7) | 25 (4.3) | 75 (7) | 63 (10.9) |
|  | **p-values** | | |  |  |  |
|  | **MSL** | **WGL** | **CL** |  |  |  |
| **ROLL** | 0.56 | 0.001 | <0.0001 |  |  |  |
| **MSL** |  | 0.03 | <0.0001 |  |  |  |
| **WGL** |  |  | 0.006 |  |  |  |

Values are n (%). ROLL= Radioguided Occult Lesion Localization; MSL= Magnetic Seed; WGL= Wire Gide Localization; CL= Carbon Localization.

**Supplementary Table S8** Surgery time including axillary management and relative p-values

| **Characteristics** | | | **ROLL** | | | | **MSL** | **WGL** | **CL** |
| --- | --- | --- | --- | --- | --- | --- | --- | --- | --- |
| **Surgery time (min)*** | | |  | | | |  |  |  |
| BCS with SLNB* | | | 60 (22) [25-189] | | | | 57 (25) [18-180] | 95 (38) [40-225] | 75 (40) [19-180] |
| BCS with ALND* | | | 100 (45) [50-212] | | | | 87 (54) [30-180] | 143 (80) [95-218] | 100 (70) [39-238] |
| **p-values** | | | | | | | |  |  |
|  | **Surgery time (BCS with SLNB)** | | | | **Surgery time (BCS with ALND)** | | |  |  |
|  | **MSL** | **WGL** | | **CL** | **MSL** | **WGL** | **CL** |  |  |
| **ROLL** | 0.01 | <0.0001 | | <0.0001 | 0.29 | 0.02 | 0.98 |  |  |
| **MSL** |  | <0.0001 | | <0.0001 |  | 0.01 | 0.35 |  |  |
| **WGL** |  |  | | <0.0001 |  |  | 0.14 |  |  |

Values are n (%) unless otherwise indicated; values * are median (i.q.r.) [range]. ROLL= Radioguided Occult Lesion Localization; MSL= Magnetic Seed; WGL= Wire Gide Localization; CL= Carbon Localization; BCS= Breast-conserving surgery; SLNB= sentinel lymph-node biopsy; ALND= axillary lymph-nodes dissection.

**Supplementary Table S9** Correlation between surgical margins involvement, localization techniques and different variables (only B5a/B5b/C5 lesions)

|  | **Nodule** | | | | | **Microcalcification** | | | | |
| --- | --- | --- | --- | --- | --- | --- | --- | --- | --- | --- |
| **Surgical margins** | **ROLL** | **MSL** | **WGL** | **CL** | **p** | **ROLL** | **MSL** | **WGL** | **CL** | **p** |
| **Not involved** | 404 (99) | 289 (96) | 665 (95.7) | 270 (91.5) | <0.0001 | 211 (94.2) | 105 (90.5) | 236 (91.5) | 147 (85.5) | 0.03 |
| **Involved** | 4 (1) | 12 (4) | 30 (4.3) | 25 (8.5) |  | 13 (5.8) | 11 (9.5) | 22 (8.5) | 25 (14.5) |  |
|  | **Distorsion** | | | | | **BMI <30** | | | | |
|  | **ROLL** | **MSL** | **WGL** | **CL** | **p** | **ROLL** | **MSL** | **WGL** | **CL** | **p** |
| **Not involved** | 42 (89.4) | 34 (89.5) | 77 (91.7) | 52 (92.9) | 0.89 | 511 (96,4) | 226 (92,2) | 845 (94,5) | 277 (89,9) | 0.001 |
| **Involved** | 5 (10.6) | 4 (10.5) | 7 (8.3) | 4 (7.1) |  | 19 (3,6) | 19 (7,8) | 49 (5,5) | 31 (10,1) |  |

Values are n (%). ROLL= Radioguided Occult Lesion Localization; MSL= Magnetic Seed; CB= core biopsy; WGL= Wire Gide Localization; CL= Carbon Localization; BMI = Body mass index

**Supplementary Table S10** Calculated Resection Ratio analysis

|  | | | **CRR** | | | | | | | | | | | | | | | |
| --- | --- | --- | --- | --- | --- | --- | --- | --- | --- | --- | --- | --- | --- | --- | --- | --- | --- | --- |
|  | | | **ROLL** | | | | **MSL** | | | | **WGL** | | | | **CL** | | | |
| **Invasive carcinoma (CB)** | | | 2.7 (4.1) [1-60.3] | | | | 2.4 (2.9) [1-60.8] | | | | 2.5 (3.2) [1-43.7] | | | | 3 (3.3) [1-40.3] | | | |
| **In situ carcinoma (CB)** | | | 2.9 (7.2) [1-72.3] | | | | 3.7 (5.7) [1-24.3] | | | | 1.5 (2.6) [1-17.1] | | | | 2.5 (4) [1-36.5] | | | |
| **Uncertain lesion (CB)** | | | 2.1 (4.4) [1-18.7] | | | | 4.4 (7.4) [1-20.6] | | | | 1.2 (0.5) [1-2.6] | | | | 1 (0.5) [1-1.5] | | | |
| **BMI <30** | | | 2.7 (5.1) [1-77.7] | | | | 1.8 (2.1) [1-14.8] | | | | 2.1 (2.8) [1-43.7] | | | | 2.7 (3.3) [1-40.3] | | | |
| **BMI ≥30** | | | 3 (6.6) [1-72.3] | | | | 3.2 (2.6) [1-24.8] | | | | 4.3 (5) [1-30] | | | | 3.8 (3.5) [1-49.9] | | | |
| **Nodule** | | | 2.2 (2.7) [1-77.8] | | | | 2 (2.3) [1-29.6] | | | | 2.4 (2.9) [1-21.5] | | | | 2.9 (3.3) [1-36.5] | | | |
| **Microcalcification** | | | 4 (7.4) [1-72.3] | | | | 4.3 (5.6) [1-90.7] | | | | 2.5 (4.8) [1-31.6] | | | | 3.3 (4.4) [1-49.9] | | | |
| **Distorsion** | | | 2.1 (5.3) [1-32.8] | | | | 3 (2.4) [1-60.8] | | | | 1.8 (2.4) [1-43.7] | | | | 3.1 (3.1) [1-22] | | | |
| **p-values** | | | | | | | | | | | | | | | | | | |
|  | **Invasive carcinoma (CB)** | | | **In situ carcinoma (CB)** | | | | **Uncertain lesion (CB)** | | | | **BMI <30** | | | | **BMI ≥30** | | |
|  | **MSL** | **WGL** | **CL** | **MSL** | **WGL** | **CL** | | **MSL** | **WGL** | **CL** | | **MSL** | **WGL** | **CL** | | **MSL** | **WGL** | **CL** |
| **ROLL** | 0.92 | 0.73 | 0.02 | 0.86 | 0.009 | 0.80 | | 0.98 | 0.60 | 0.22 | | <0.0001 | <0.0001 | 0.97 | | 1.00 | 0.33 | 0.44 |
| **MSL** |  | 1.00 | 0.002 |  | 0.19 | 1.00 | |  | 0.86 | 0.36 | |  | 0.08 | <0.0001 | |  | 0.20 | 0.34 |
| **WGL** |  |  | <0.0001 |  |  | 0.009 | |  |  | 0.94 | |  |  | 0.003 | |  |  | 0.91 |
|  | **Nodule** | | | **Microcalcification** | | | | **Distorsion** | | | |  |  |  | |  |  |  |
|  | **MSL** | **WGL** | **CL** | **MSL** | **WGL** | **CL** | | **MSL** | **WGL** | **CL** | |  |  |  | |  |  |  |
| **ROLL** | 0.80 | 0.37 | <0.0001 | 1.00 | <0.0001 | 0.20 | | 0.84 | 0.99 | 0.22 | |  |  |  | |  |  |  |
| **MSL** |  | 0.06 | <0.0001 |  | 0.002 | 0.38 | |  | 0.37 | 0.82 | |  |  |  | |  |  |  |
| **WGL** |  |  | 0.004 |  |  | 0.09 | |  |  | 0.02 | |  |  |  | |  |  |  |

Values are median (i.q.r.) [range]. CRR = Calculated resection ratio; ROLL= Radioguided Occult Lesion Localization; MSL= Magnetic Seed; CB= core biopsy; WGL= Wire Gide Localization; CL= Carbon Localization; CB = Core biopsy; BMI = Body mass index

**Supplementary Table S11** Hierarchical Logistic Regression Modeling. The effect of each center in multivariate analysis.

| **Center** | **p** |
| --- | --- |
| **IRCCS Azienda Ospedaliero-Universitaria - Bologna** | 0.35 |
| **ASST Sette Laghi - Varese** | 0.24 |
| **EOC - Svizzera** | 0.96 |
| **Humanitas Gavazzeni - Bergamo** | 0.50 |
| **Istituti Clinici Scientifici IRCCS - Pavia** | 0.27 |
| **Istituto Nazionale dei Tumori - Milano** | 0.37 |
| **I.R.C.C.S. Fondazione G. Pascale - Napoli** | 0.53 |
| **Azienda Ospedaliero Universitaria - Modena** | 0.30 |
| **Fondazione IRCCS Policlinico San Matteo - Pavia** | 0.96 |
| **VDE e AVC – P. O. - Pontedera e Volterra** | 0.05 |
| **Ospedale Santa Chiara - Trento** | 0.22 |
| **ASST Fatebenefratelli Sacco - Milano** | N/A |
| **Azienda Sanitaria n.1 Regione Umbria - Città di Castello** | N/A |

**Supplementary Table S12** Multivariate analysis excluding B3/B4 lesions

|  | **Computed the probability of surgical margins involvement** | | |
| --- | --- | --- | --- |
|  | **OR** | **95%CI** | **p-value** |
| **Localization techniques** |  |  |  |
| CL | 1.84 | 1.22-2.76 | 0.003 |
| MSL | 0.89 | 0.48-1.63 | 0.70 |
| ROLL | 0.48 | 0.26-0.89 | 0.02 |
| WGL | Ref. |  |  |
| **Surgeon who performed resection** |  |  |  |
| Attending surgeon | 1.83 | 0.79-4.24 | 0.16 |
| Resident | Ref. |  |  |
| **Lesion morphology** |  |  |  |
| Microcalcification | 1.82 | 1.02-3.24 | 0.04 |
| Distorsion | 2.12 | 1.4-3.19 | 0.0004 |
| Nodule | Ref. |  |  |
| **Lesion size on imaging (mm)** | 1.01 | 1.00-1.03 | 0.05 |
| **CRR** | 0.99 | 0.95-1.02 | 0.44 |

**References**

1. Krekel NMA, Zonderhuis BM, Stockmann HBAC, Schreurs WH, van der Veen H, de Lange de Klerk ESM, et al. A comparison of three methods for nonpalpable breast cancer excision. European Journal of Surgical Oncology (EJSO). 2011 Feb;37(2):109–15.
